# Medical insurance, health risks, and household financial asset allocation: evidence from China household finance survey

**DOI:** 10.3389/fpubh.2023.1268470

**Published:** 2023-12-22

**Authors:** Chengming Li, Jiashan Li, Chenchen Zhai, Xiaoqi Dong, Zhengyu Jiang, Shaoxiang Jiang

**Affiliations:** ^1^School of Economics, Minzu University of China, Beijing, China; ^2^China Institute for Vitalizing Border Areas and Enriching the People, Minzu University of China, Beijing, China; ^3^Department of Applied Mathematics and Statistics, Stony Brook University, Stony Brook, NY, United States; ^4^School of Economics and Finance, Xi'an Jiaotong University, Xi'an, China; ^5^Institute for Global Health and Development, Peking University, Beijing, China

**Keywords:** medical insurance, health risk, household finance, asset allocation, China household finance survey

## Abstract

There is a lack of micro evidence on whether medical insurance may optimize the household financial asset allocation by transferring health risk, despite the fact that health risk is a significant component driving families’ precautionary savings. This article empirically examines the impact of health risk and social medical insurance on household risky financial asset allocation using a Probit model, based on data from the 2015–2019 China Household Finance Survey (CHFS). The findings indicate that social medical insurance, with its lower level of security, reduces the likelihood, but it can alter households’ preferences for risk by lowering marginal effect of health risk. According to the findings of the heterogeneity analysis, people who live in rural and less developed areas are more likely to experience the risk-inhibiting effects of social medical insurance and health risk. The eroding and risk-suppressing impacts of social medical insurance are likewise less pronounced for households headed by women and older people, as is the health risk’s suppressive influence on household involvement in risky financial markets. Compared with social medical insurance, commercial medical insurance with a higher level of coverage can dramatically increase household participation in riskier financial markets. This article provides micro-empirical evidence for the household asset allocation effect of medical insurance.

## 1 Introduction

Due to advancements in technology, improvements in healthcare, and concerns related to viral transmission, global attention to health issues is continuously growing. However, the health status of communities varies significantly from one nation to another based on factors such as education, income, geographic location, ethnicity, and gender. The World Health Organization has emphasized the need to focus on the most vulnerable segments of society to improve overall health. Additionally, health risk is a crucial contextual factor that influences how households allocate their financial assets and, consequently, the nation’s economy (1). Research by Atella et al. (2) has indicated that medical insurance, fundamental insurance like social medical insurance, plays a significant role in mitigating health risks and shaping the distribution of household financial assets. Therefore, it is essential to carefully consider the impact of medical insurance and formulate effective policies to encourage beneficial interactions between the medical insurance system and household finances. It is noteworthy that there is currently a lack of research examining the influence of health risk and social medical insurance on household financial asset allocation.

Financial asset allocation is the process of allocating an investor’s money across various financial assets (such as stocks, bonds, deposits, etc.) based on their individual needs. According to asset portfolio theory, households will decide the investment ratio between risky and risk-free assets based on their individual risk preferences under the assumption of a rational economic person (3). Households make investment decisions by balancing the risks they encounter against the returns when determining the kind and amount of financial assets to invest in. The majority of current research on the variables affecting household financial asset allocation focuses on life-cycle theory (4, 5), demographic features (6–9), and life-cycle theory. There is less literature that combines the level of health coverage and considers health risks comprehensively. Regarding the relationship between risk preferences and household financial asset allocation, according to Markowitz’s (3) modern portfolio theory, investors generally allocate risky financial assets according to their risk preferences. Tobin (10) further states that investors’ risk attitudes (risk averse, risk neutral, and risk favored) determine their level of investment risk preferences. The more risk-averse an investor is, the lower the probability of investing in risky financial assets and the lower the proportion of risky financial assets allocated (or vice versa) (11). Therefore, this article argues that family members with higher risk appetites will have a higher probability of investing in a greater proportion of risky financial assets. Also, households with more risky financial assets imply that members of this family have higher risk appetites.

In the field of research on the relationship between medical insurance and household asset choice, there are two types of views. One scholars believes that medical insurance can increase policyholders’ willingness to hold risky assets and increase risky investments (12–14); The opposing viewpoint holds that because diverse medical insurance security levels exist, some medical insurance does not significantly improve financial security, and as a result, it cannot effectively promote household participation in risky financial markets. Li (15) and others find that medical insurance with higher security levels is more likely to promote household risky financial asset holdings. According to Goldman and Maestas (13), higher levels of household medical insurance security are associated with an increased likelihood of investing in risky financial assets.

Health risk, which refers to the overall health risk of the household, is a significant factor affecting the allocation of financial assets within households, exerting both direct and indirect influences. From a direct impact perspective, medical insurance provides financial security to address unforeseeable health events, thereby transferring health risks. However, health risks can lead to increased medical expenses, consequently reducing the wealth of households (16), directly affecting their financial situation. Particularly for individuals with poorer health conditions, financial stability becomes more crucial, prompting a preference for cautious savings to safeguard against unexpected medical costs (13, 17). Additionally, they tend to adopt a more conservative approach in investment, favoring low-risk financial assets (1) to maintain financial security. On the other hand, health risk indirectly affects a household’s earning capacity as health issues can diminish the labor force. High-income households are more inclined to engage in riskier financial markets and hold a more significant proportion of high-risk financial assets (18). Thus, an increase in income can enhance a household’s risk preference, leading to the selection of more perilous financial assets (19). Consequently, health risk indirectly shapes the allocation of financial assets by influencing a household’s income-generating potential.

Compared to prior research, this article offers four significant contributions:

Firstly, from a research perspective, this paper broadens the scope of inquiry within the field of household financial asset allocation. Previous literature has focused more on life cycle (4, 20, 21), financial literacy (22–26), household asset size (27, 28), et cetera, with less literature considering health risk as a background risk and social medical insurance in combination. This paper selects social medical insurance in general for analysis, considers health risk as an important background risk, and introduces the concept of risk constraint effect. The differential impact of social medical insurance and commercial medical insurance with different levels of coverage is further discussed, bridging a gap in the relevant research area and providing some literature reference value for the intersection of social security, medical insurance, and financial asset allocation. In medical insurance, previous literature has predominantly examined its effects on consumption and healthcare expenditures, with relatively limited exploration of its impact on household financial asset allocation. Previous literature has more often studied its impact on consumption (29), and medical expenditures (30, 31), and less often on household financial asset allocation. Moreover, the choice of the type of medical insurance is also mainly focused on the subdivision of social medical insurance, and basic medical insurance (32, 33), and less literature has studied social medical insurance in general and taking health risks into account. Even less research has been done contrasting the effects of social and commercial medical insurance, two insurance systems with varying levels of coverage. This article examines the effects of social medical insurance generally on the financial asset allocation of households as well as how it affects health risk. It serves as a resource for further investigation in connected fields.

Secondly, concerning research content, prior studies have shown limited attention to medical insurance and health risk, and there is a dearth of literature considering the marginal effects of medical insurance on health risk. This article examines how social medical insurance affects the marginal impact of health risk. It offers fresh perspectives for future researchers’ work on background risk and medical insurance.

Additionally, in terms of research methodology, this article employs the instrumental variable approach to address endogeneity concerns. The instrumental variable method is highly applicable and adept at handling a wide range of endogeneity issues, thereby establishing a foundational resource for addressing endogeneity concerns in subsequent research pertaining to household financial asset allocation.

Finally, concerning the research’s broader significance, this article delves into the factors influencing the financial asset allocation of Chinese households. It contributes to formulating evidence-based insurance and financial policies in high-volume developing countries. Furthermore, it bears crucial implications for households in making informed decisions regarding insurance and financial asset allocation.

**Figure 1:**
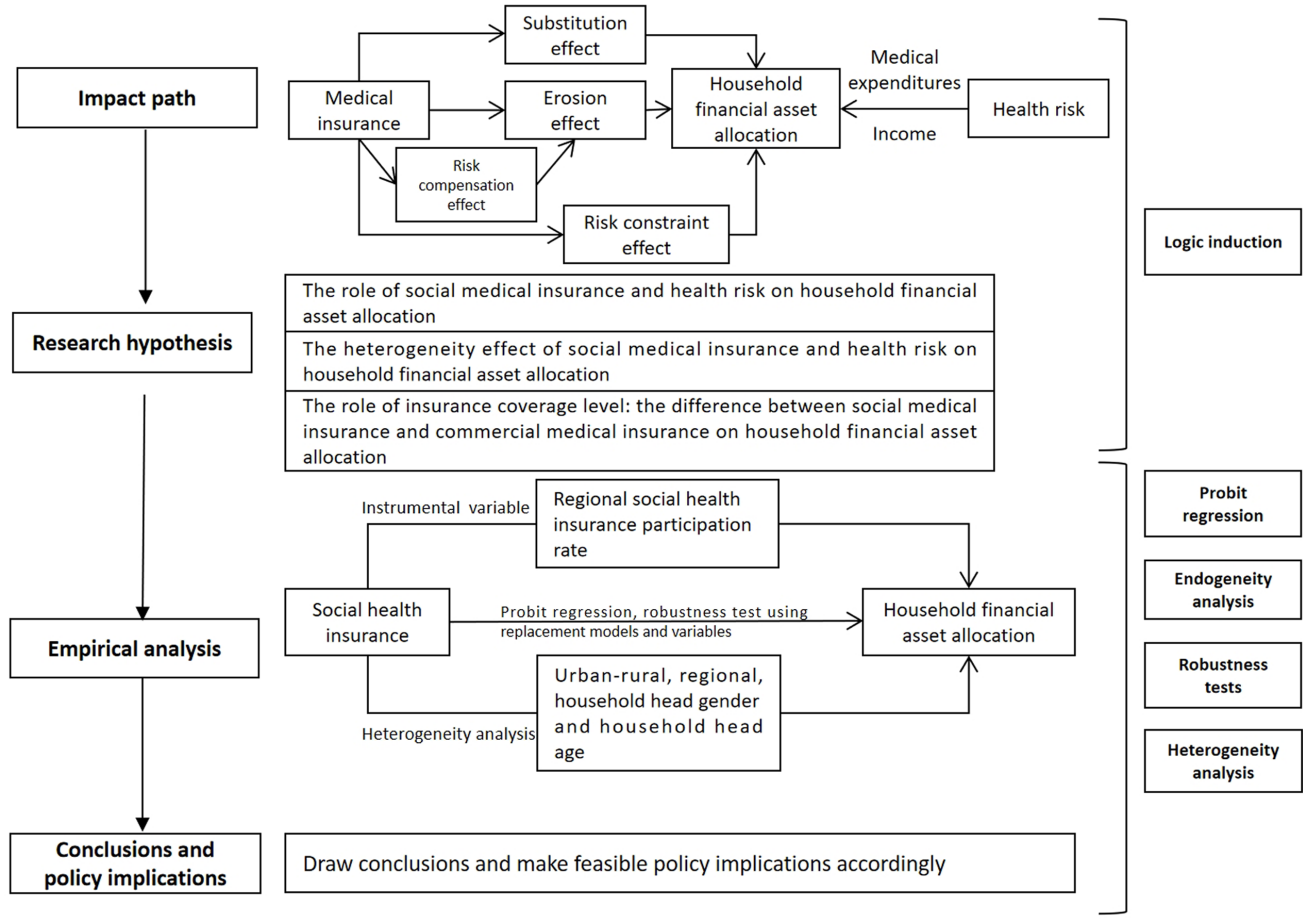
The framework of this article.

The rest of the article is organized as follows: Section 2 presents the theoretical analysis and research hypotheses; Section 3 presents the research design, introducing the model design, data sources, variable descriptions, and descriptive statistics; Section 4 analyzes the baseline regression results of medical insurance on household financial asset allocation, conducts an endogeneity analysis using instrumental variables, and performs a series of robustness tests on the model and empirical results. In Section 5, the article further discusses the differential effects of urban–rural, regional, gender of household head, education level of household head, and age of household head; Section 6 further discusses the differential effects of two insurance systems with different levels of coverage, commercial medical insurance and social medical insurance, on household risk financial asset allocation; Section 7 summarizes the entire article and makes relevant policy implications (Figure 1).

## 2 Theoretical analysis and research hypothesis

Through a review of the literature, this article concludes that there are four main ways in which medical insurance affects financial asset allocation: the substitution effect, the erosion effect, the risk compensation effect, and the risk constraint effect. The substitution effect refers to the effect that medical insurance can compensate all or part of a household’s medical expenses after a health risk occurs, thus increasing the household’s investment risk appetite (34). The erosion effect refers to reducing household wealth and assets because of the increase in health care expenditures that households make in order to pay for medical insurance. In addition, households are likely to increase health care demand and medical consumption after paying for medical insurance, which may lead to over-medication and potentially increase the burden of health care (35). As a result, households may have lower disposable income due to premiums or excessive medical expenses, which may reduce their risk appetite and reduce their investment in risky financial assets. The risk compensation effect refers to the fact that insurance companies face adverse selection and moral hazard problems due to information asymmetry (36–38), thus increasing risk premiums and raising premiums by considering the insurance company’s own risk factors when pricing insurance. Higher premiums increase the erosion effect, which discourages households’ risk preferences. The risk constraint effect is the effect of medical insurance that reduces health risks and their adverse effects.

The combined effect of the substitution and erosion effects of medical insurance is related to the extent of medical insurance coverage. “Medical insurance coverage” refers to the reimbursement levels within a medical insurance plan. Medical insurance cannot considerably improve household risk appetite and increase the share of hazardous financial assets allocated if the level of coverage is low and there is no method to pay for significant medical expenses. Some scholars (33, 38, 39) have found that both urban workers’ medical insurance and new rural cooperative medical insurance in basic medical insurance increase the household medical burden. Therefore, it can be clearly stated that medical insurance with different coverage levels will hurt the distribution of household financial assets. High-coverage medical insurance may raise the percentage of risky household financial assets. As a result of higher resident medical costs and lower predicted risk compensation, social medical insurance with a lower level of coverage reduces the household’s hazardous financial assets.

### 2.1 The role of social medical insurance and health risk on household financial asset allocation

Health risk is a heavily weighted background risk in households and can affect household financial asset allocation. Health risk itself cannot be distributed across time, unlike asset risk, and influences on how people choose their assets. Health risk causes unexpected increases in medical costs and has an impact on family members’ capacity to earn a living, which lowers household income (40). In order to manage the uncertain costs associated with health risks, this is therefore primarily manifested from the perspective of household financial asset choice as increased precautionary savings (41), increased risk-free asset holdings, and decreased risky asset holdings (8). Families have the option of either retaining or transferring risks when it comes to managing health concerns. Retaining risk entails that the household is responsible for covering all unforeseen costs, and the success of this strategy is based on the wealth and income of the household. For most average families, high medical expenses could easily lead to a financial crisis. The main means of transferring risk is currently the purchase of medical insurance.

Social medical insurance can reduce health risks and their effects. Some scholars argue that the medical field is essential to residents’ healthy lives (42) in this context, medical insurance can improve residents’ health conditions by reducing medical costs, enhancing healthcare services, and increasing the quantity and quality of medical services purchased by residents (43). Thus, on the one hand, medical insurance can reduce health care costs and effectively improve the accessibility of health care services (44), promoting more health care services for insured households and thus improving their own health capital (45). On the other hand, insured individuals will increase their health concerns and improve their health capital by reducing health-harming behaviors and increasing health care expenditures. This leads to a reduction in the likelihood of illness or deterioration of illness among the middle-aged and older adult or chronically ill, further reducing the large medical expenditures due to health risks, weakening the effect of health risks on the risk aversion of household financial assets and promoting the release of more liquidity for households to invest in risky financial assets. Although social medical insurance has limited coverage for major diseases, it plays a vital role in promoting participants’ daily health care and improving health service utilization. Medical insurance can improve the health status of the insured by promoting the health concerns of families and increasing the motivation to prevent and treat minor illnesses. Therefore, medical insurance can effectively reduce health risks and its marginal impact on household financial asset allocation. Accordingly, it is proposed that:

*H1*: Social medical insurance can lessen the marginal effect of health risk on the distribution of household financial assets.

### 2.2 The heterogeneity effect of social medical insurance and health risk on household financial asset allocation

In terms of household income level, insurance philosophy, and level of health care services, there are differences between urban and rural areas, regions, and households headed by different genders, educational levels, and ages. As a result, there are differences in how social medical insurance and household health risks affect the structure of household financial asset allocation. There are differences in income between urban and rural areas and regions. At the same time, most studies conclude that the higher the income, the higher the participation in the risky financial asset market (18). Therefore, for households in urban and developed areas, there tends to be a higher willingness to participate in risky financial markets, with smaller impact coefficients for health risks and medical insurance. Due to gender differences, there can be natural differences in risk preferences between men and women, with women generally being more risk averse (46), and thus male household heads are more likely to choose risky financial assets such as stocks than women (7). Female heads of households have a lower willingness to participate in risk markets and are less affected by health risks and medical insurance. Additionally, the life-cycle hypothesis contends that as people become older, their asset preferences shift, and numerous academics have used microdata to confirm this claim empirically. Retirement is a crucial life stage that impacts household asset decisions and has the potential to affect participants’ willingness to take risks in the financial markets. According to several studies, investors who are retired are more risk-averse (47). Because of this, households headed by people over 60 tend to be naturally risk cautious and prefer low-risk financial products, which are less influenced by health risks and medical insurance.

*H2*: The erosion effect and risk inhibition effect of social medical insurance are more pronounced for inhabitants of rural and less developed areas, where health risks have a more significant inhibitory influence on household risk financial market involvement. The erosion impact and risk inhibition effects of social medical insurance are smaller for households headed by women and those headed by older adults. Health hazards also have a weaker inhibitory effect on household risk financial market involvement.

### 2.3 The role of insurance coverage level: the difference between social medical insurance and commercial medical insurance on household financial asset allocation

Most scholars believe that significant medical expenses will burden the family economy and worsen the family’s financial situation (48). By utilizing medical insurance subsidies, families who purchase medical insurance can achieve risk transfer and lower family economic risk (49). When family members are at risk for health issues, the financial support provided by medical insurance can lessen the effect that unforeseen medical costs will have on the family’s financial situation and even enhance it (33). As a result, households may reduce precautionary savings (50) and increase their holdings of risky assets (49, 51). However, because social and commercial medical insurance offer varying levels of protection, the distribution of family financial assets will also have a difference in impact. The amount that unexpected family medical expenses can be covered by insurance with less protection is constrained. The level of social medical insurance is generally minimal, making it ineffective at relieving families’ burden of high medical costs and having a poor substitution effect. More often than not, social medical insurance just encourages medical consumption and expenditure. When combined with the regular rises in social medical insurance premium costs, it will lessen households’ inclination to invest in risky financial assets. Commercial medical insurance offers a higher level of security and can pay for significant unforeseen medical costs. The families that get commercial medical insurance also frequently earn more money, the premium-to-income ratio is low, the erosion effect is minimal, and the substitution effect is significant. Because of this, households covered by commercial insurance may reduce their precautionary savings and raise their holdings of hazardous financial assets once the risk of substantial medical expenses has been adequately reduced.

*H3*: Due to the different levels of medical insurance coverage, social medical insurance will reduce the probability of household allocation to risky financial assets, while commercial medical insurance will promote household participation in risky financial markets.

## 3 Model, data and variables

### 3.1 Model design

In order to examine the impact of health risk and medical insurance on household financial asset allocation, the following econometric models is set in this article:


(1) $$\begin{array}{l} \mathrm{riskasse}t_{i}=\beta +\beta_{1}\mathrm{insuranc}e_{i}+\beta_{2}\mathrm{healthris}k_{i}\\ \;+\;\beta_{3}\mathrm{insuranc}e_{i}\times \mathrm{healthris}k_{i}+\gamma_{1}X_{i}+\varepsilon_{i} \end{array}$$

In the formula, *risk asset_i_* is a binary variable representing whether to hold risky financial assets, taking values of 1 and 0. The core explanatory variables are *healthrisk_i_*, whether to participate in medical insurance *insurance_i_*. and the interaction term of social medical insurance and health risk *insurance_i_* × *healthrisk_i_*. *X_i_* is a control variable, including control variables of household characteristics and control variables of individual characteristics. *ε_i_* is an unobservable error term.

### 3.2 Data sources

The panel data used in this article were created by matching household questionnaires with individual questionnaires using household codes and individual codes from the China Household Financial Survey (CHFS) of the China Household Financial Survey and Research Center, which was conducted from 2015 to 2019. This data sample spans 29 provinces in China (excluding Tibet, Xinjiang, Hong Kong, Macao, and Taiwan) as well as 367 districts and counties. The database examines information on Chinese households’ purchasing patterns, income levels, debt loads, health status, and other factors. A total of 34,603 home samples were gathered after data processing. The data in this article are mostly processed as follows: (1) The samples missing important codes, such as household, individual, urban–rural, and provincial codes, which cannot be processed by the interpolation approach, are eliminated from the analysis in this work. (2) Zero is allocated to the missing values in this article’s variables for holding risky financial assets, social pension insurance type, medical expenses, and accounts receivable. This is because while answering these questions, the respondents frequently omit their responses due to a lack of relevant asset allocation information or relevant expenditure data. In this situation, it would be more logical to substitute a value of 0 for the missing data.

### 3.3 Variable selection

The primary factors we concentrate on in this article are residents’ social medical insurance health status, and household hazardous financial asset allocation. Additionally, these elements are added as control variables in the sample regression in order to account for the influence of demographic features. Data collection is conducted at the individual level and subsequently summarized and analyzed at the household level. We allocate a unique identification code to each household for statistical purposes. As long as at least one member of a household participates in the insurance plan, it signifies that the entire household is covered by insurance. The following is a description of the variables chosen and their definitions:

#### 3.3.1 Explained variables

This article examines the distribution of household financial assets with a focus on whether households invest in risky financial instruments and the extent of their involvement in the risky financial market. To do this, two variables—whether they do so and the percentage of risky financial assets—are chosen. This article characterizes risky financial assets as all financial assets other than cash, bank demand deposits, and time deposits, drawing on the body of current work. Due to the possibility of bad debts, household lending is likewise categorized in this article as a risky financial asset. If a household has one or more risky financial assets, it is deemed to be investing in these assets and is given a value of 1, otherwise it is given a value of 0.

The subjective question of whether residents are willing to invest in hazardous financial assets is substituted for the objective indicator of whether the explanatory variable in the benchmark regression holds risky financial assets in the robustness check section. The indicator of willingness to invest in risky financial assets is derived from the investment risk preference question designed in the CHFS questionnaire: If you had a sum of money to invest, which investment item would you be most willing to choose? The answers include: 1. high risk, high return project; 2. slightly higher risk, slightly higher return project; 3. average risk, average return project; 4. slightly lower risk, slightly lower return project, and 5. unwilling to take any risk. According to the respondents’ answers to the above questions, when the respondents choose 1, 2, 3, or 4, a value of 1 is assigned, indicating that the residents are willing to invest in risky financial assets; when the respondents choose 5, a value of 0 is assigned, indicating that the residents are not willing to invest in any form of risky financial assets (Table 1).

**Table 1:** Delineation of risky financial assets and risk-free financial assets.

Name of the asset	Delineation of criteria
Risky financial assets	Stocks, bonds, funds, financial derivatives, gold and other precious metals, financial products, Internet financial products, foreign exchange, lending money, etc.
Risk-free financial assets	Cash, bank demand deposits, bank time deposits

#### 3.3.2 Explanatory variables

The core explanatory variables in this article are social medical insurance, health risk, and the interaction term of social medical insurance and health risk. For social medical insurance, this article utilizes the CHFS questionnaire data on household participation in social medical insurance. This article also uses the household health risk rate and the household social insurance participation rate to replace the two variables of household health risk and participation in social insurance for robustness testing. By dividing the number of household participants by the total number of household participants, the household commercial medical insurance participation rate and the household social medical insurance participation rate were determined. The values were between 0 and 1.

This article takes into account the fact that households with poor health status have a higher health risk. In order to quantify the health risk of families using the family self-rated family health risk, the self-rated health status of family members is chosen in this article. There are five categories of health status in the CHFS questionnaire: very good, good, fair, bad, and very bad. These categories are represented by the numbers 1, 2, 3, 4, and 5 accordingly. In this article, poor physical health status is defined as average, bad, or very bad, and is given a value of 1, while good physical health status is defined as very good or good, and is given a value of 0. Based on this, this article calculates the number of poor health status of a household as a unit, which is used to measure and indicate the size of health risk existing in the household. The interaction term between health risk and social medical insurance is generated to verify the impact of medical insurance on household financial asset allocation through the mediator of health risk.

#### 3.3.3 Control variables

In this article, personal characteristics and household characteristics control variables are selected. The control variables of household characteristics mainly include: the number of household size, total household income, and medical expenditure; the individual variables mainly include: the number of years of education of the household head, age, gender, and the type of social pension insurance.

*Number of household members*: Households with large household members will face more cost of living and greater risk factors. Therefore, household consumption and investment behavior will receive the influence of household size.

*Total household income*: Households with higher household income will have more liquidity, which may affect their investment behavior. Households with higher incomes are more likely to participate in risky financial markets. In this article, total income is added to the econometric model as a control variable after taking the logarithm of the total income.

*Years of education*: Education affects one’s investment perceptions, and investors with more financial knowledge tend to use their portfolios to increase returns and therefore have a greater probability of participating in risky financial markets. In this article, years of education are calculated based on a survey of household members’ education levels in a questionnaire.

*Age*: The investment profile of households receives life-cycle effects, older households tend to be risk-averse and hold more robust assets, such as: cash, savings, and other assets. In this article, the age variable is obtained by making an inference based on the birth time of the household head in the questionnaire and involving the square of age in the regression.

**Figure 2:**
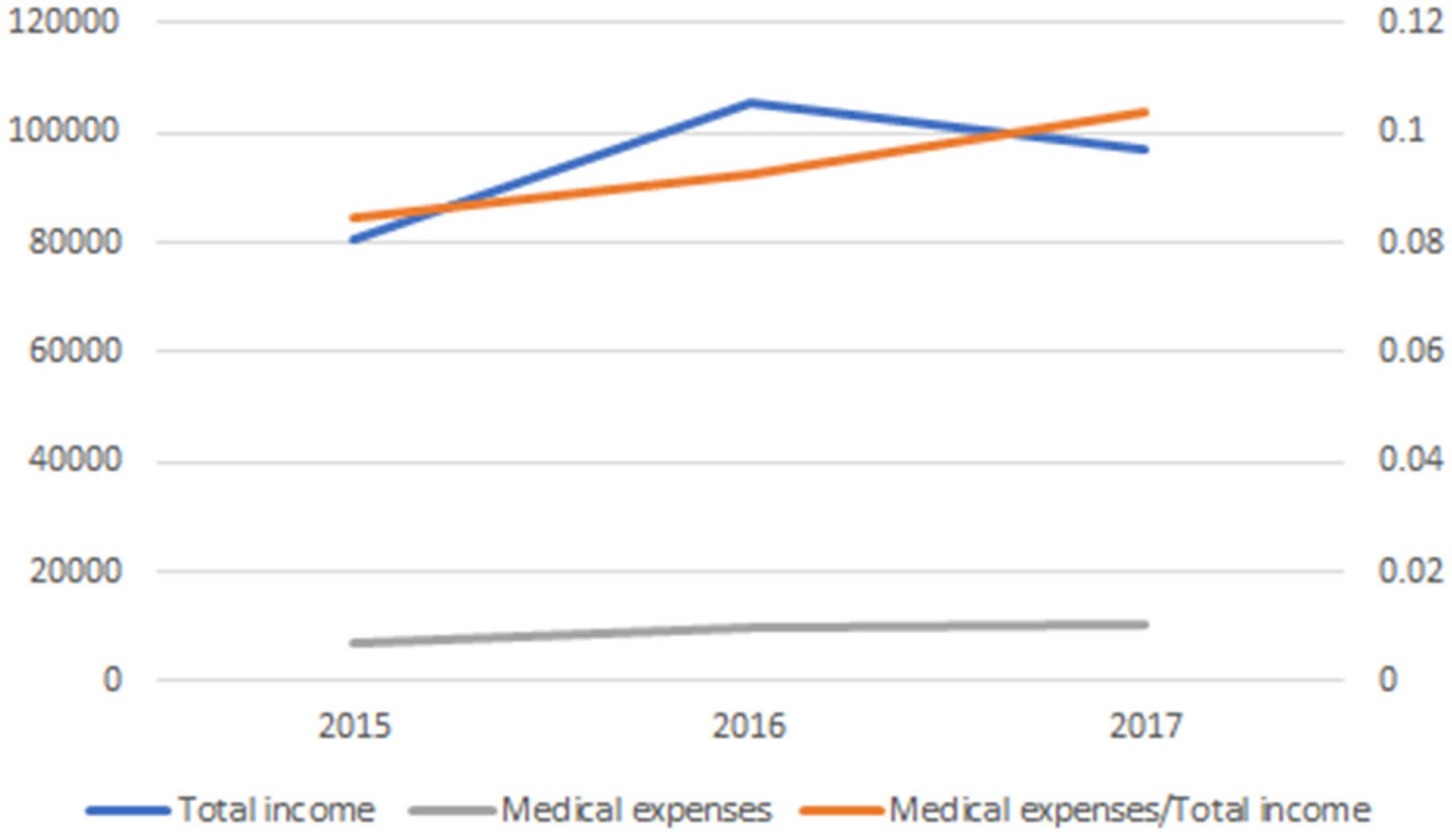
Medical expenses trend chart.

*Medical expenditure*: Healthcare expenditure has become the third-largest household expense, following housing and education expenditures. This trend has attracted the interest of many scholars for research (52). Medical expenditure is something that increases the financial burden of households and increases their financial vulnerability, so households with higher medical expenditure are likely to be more risk-averse, regardless of other factors. From Figure 2, it can be observed that household medical expenses have been steadily rising year by year, and their share of total household income has also been increasing annually.

*Gender*: Gender affects investment preferences.

*Type of social pension insurance*: Social pension insurance provides livelihood security for residents after retirement, and families may be more inclined to invest in risky financial assets (Table 2).

**Table 2:** Variable description.

Variable description	Definition
Explained variables	
Risky financial assets	Financial assets other than cash and bank savings deposits are considered as risky financial assets. According to the CHFS questionnaire survey, if a household holds any form of risky financial assets, it is coded as 1; otherwise, it is coded as 0.
Willingness	Willingness to invest in risky financial assets. Using the investment risk preference question designed in the CHFS questionnaire: If you had a sum of money to invest, which investment project would you be most willing to choose? The answers include: 1. high risk, high return project; 2. slightly high risk, slightly high return project; 3. average risk, average return project; 4. slightly low risk, slightly low return project, and 5. unwilling to take any risk. According to the respondents’ answers to the above questions, when the respondents choose 1, 2, 3, or 4, a value of 1 is assigned, indicating that the residents are willing to invest in risky financial assets; when the respondents choose 5, a value of 0 is assigned, indicating that the residents are not willing to invest in any form of risky financial assets.
Explanatory variables	
Social medical insurance	Whether to participate in social medical insurance. 1 if participating, 0 if not participating
Health risks	The health risk was measured by whether the physical condition was poor, with poor physical condition, health risk of 1, and good physical condition, health risk of 0. With the help of the question in the CHFS questionnaire: How is the physical condition now compared to the peers? The responses were designed as 1. very good; 2. good; 3. average; 4. bad; and 5. very bad, where respondents who chose 3, 4, and 5 were in poor physical condition and assigned a value of 1, and respondents who chose 1 and 2 were in good health and assigned a value of 0.
Household health risks *social medical insurance	Interaction term between health risk and social medical insurance. The number of people with poor health status in the household is calculated as a unit, which is used to measure and express the magnitude of health risk that exists in the household, and then multiplied with whether or not to participate in social medical insurance
Household health risk rate	Household health risk rate = number of people in poor physical condition in the household/number of people in the household
Household social medical insurance participation rate	Family social medical insurance participation rate = number of family participants in social medical insurance / number of family members
Commercial medical insurance	Whether to participate in commercial medical insurance.1 if participating, 0 if not participating
Household health risks *commercial medical insurance	Interaction term between health risk and commercial medical insurance. The number of people with poor health status in the household is calculated as a unit, which is used to measure and express the magnitude of health risk that exists in the household, and then multiplied with whether or not to participate in commercial medical insurance
Risky assets ratio	Risky assets ratio = risk assets/household total assets
Control variables	
Number	Number of family members. This is the whole number, which is a count of the number of family members
ln_total income	This is a continuous variable, taking the logarithm of total household income
Education	Years of education. According to the CHFS questionnaire, the education level of household members is: 1. never attended school; 2. elementary school; 3. junior high school; 4. high school; 5. junior high school/vocational high school; 6. college/vocational high school; 7. university undergraduate 8. master’s degree 9. doctoral degree 0 for no school; 6 for elementary school; 9 for junior high school;12 for high school/junior college/vocational high school; 15 for college/vocational high school; 16 for university undergraduate; 18 for master’s degree; 22 for doctoral degree
Age	Age squared
Ln_medical expenses	This is the continuous variable, where the household’s annual medical expenditure
Gender	Male = 0, Female = 1
Social pension insurance	Types of social pension insurance. In the CHFS data, the specific questions are: which of the following social pension insurance is participated in? 5 = Organization and institution pension/retirement pension; 4 = Basic pension insurance for urban workers (urban employment insurance, generally paid monthly); 3 = New rural social pension insurance (new rural insurance, paid annually); 2 = Urban residents’ social pension insurance (urban residence insurance, paid annually); 1 = Unified urban and rural residents’ social pension insurance (annual payment); and 0 is not participated in social pension insurance.

### 3.4 Descriptive statistics

According to the statistical results of the overall data, it can be obtained that with the continuous improvement and popularization of China’s social medical insurance system, the social medical insurance participation rate of our residents is high at 0.9193. Due to the high cost of commercial medical insurance and the slow diffusion of knowledge about commercial medical insurance, the rate of holding commercial medical insurance is 0.0281, which is much lower than that of social medical insurance. The chance and proportion of allocating risky financial assets are also at a low level in China’s household financial asset allocation, with the probability of allocating risky financial assets being 0.4221 and the average value of risky assets ratio being 0.0970. The likelihood that one’s physical condition is suboptimal is 0.5018, and the average family health risk is 2.1279. The average length of education is 7.7086 years, and there is still room for improvement in this area. There is an imbalance in the gender ratio, with the female ratio being 0.4921, which is somewhat lower than the male ratio. Influenced by urbanization, the rural population ratio is 0.3762, which is much lower than the urban population ratio (Table 3).

**Table 3:** Variable descriptive statistics.

	Mean	Std.	Min.	Max.
Medical insurance	0.9213	0.26931	0	1
Social medical insurance	0.9193	0.2724	0	1
Health risk	0.5018	0.4999	0	1
Commercial medical insurance	0.0281	0.1652	0	1
Risky financial assets	0.4221	0.4939	0	1
Risky assets ratio	0.0970	0.2327	0	1
Number	4.2720	1.7761	1	20
Household health risks	2.1279	1.8423	0	19
Education	7.7086	5.3196	0	22
Age	43.0712	21.7826	5	119
Medical expenses	8,758.143	30,280.65	0	2,834,481
Gender	0.4921	0.4999	0	1
Social pension insurance	1.4528	1.4644	0	5
Ln_total income	10.3212	3.1699	−15.51902	16.31057
Rural	0.3762	0.4844	0	1

To further investigate the effect of medical insurance on household financial assets, this article presents statistics by whether households purchase risky assets or not. The statistical results show that the mean value of household health risk is 1.7444 among households that purchase risky financial assets, which is lower than 2.4079 among households that do not allocate risky financial assets; the mean value of commercial medical insurance participation among households that allocate risky assets is 0.0469, and the social medical insurance participation rate is 0.9142, which is higher and lower than 0.0143 and 0.9229 among households that do not allocate risky financial assets, respectively (Table 4). Therefore, it can be hypothesized that: 1. Health risks, in other words, poor health status reduces households’ willingness to invest in risky financial markets; 2. A higher degree of commercial medical insurance coverage increases households’ risk appetite; and 3. Social medical insurance has a limited impact on households’ risky financial asset allocation due to its limited coverage.

**Table 4:** Descriptive statistics by whether risky assets are purchased.

	Whether to hold risky assets	
	No	Yes
Health risk	0.5452	0.4424
Household health risks	2.4079	1.7444
Social medical insurance	0.9229	0.9142
Commercial medical insurance	0.0143	0.0469
Risky assets ratio	0	0.4509

The average health risk for households with medical insurance is 2.088, which is lower than the average of 2.5900 for households without medical insurance. The average health expenditure for households with medical insurance is 8,733.499, which is lower than the average of 9,046.511 for households without medical insurance. The mean value of risky financial assets as a percentage of total financial assets is 0.0973, which is higher than the mean value of 0.0924 for uninsured households, thus, it can be assumed that: firstly, participation in medical insurance will have a substitution effect, lowering medical expenditures and increasing the proportion of risky financial assets of households; Second, participation in medical insurance reduces household health risk and the impact of health risk on household risk preferences, thus promoting household allocation to risky financial assets (Table 5).

**Table 5:** Descriptive statistics by whether or not to purchase medical insurance.

	Whether to participate in medical insurance	
	No	Yes
Health risk	0.5472	0.4979
Household health risks	2.5900	2.088
Medical expenses	9046.511	8733.499
Risky assets ratio	0.0924	0.0973

In this article, the statistics are conducted by households according to whether they participate in social medical insurance or not. The results show that the mean health risk of households with social medical insurance is 0.4985 and the family health risk is 2.0902, both lower than the mean of 0.5393 and 2.5568 for households without social medical insurance; the mean risky assets ratio of households with social medical insurance is 0.0969, lower than the mean of 0.0976 for households without social medical insurance. The probability of participating in risky financial assets is 0.4198, which is also lower than that of 0.4484 for non-participating households. Thus, it can be speculated that social medical insurance can, to some extent, draw attention to health and reduce health risks. However, because of its low level of coverage and erosion effect, it can instead reduce household risk appetite (Table 6).

**Table 6:** Descriptive statistics by whether or not to purchase social medical insurance.

	Whether to participate in social medical insurance	
	No	Yes
Health risk	0.5393	0.4985
Household health risks	2.5568	2.0902
Risky financial assets	0.4484	0.4198
Risky assets ratio	0.0976	0.0969

In this article, the statistics are conducted on a household basis, according to whether or not they are covered by commercial medical insurance. The statistical results show that the health risk and family health risk of households participating in commercial medical insurance are much lower than those of households not participating in commercial medical insurance. The average health expenditure of households with commercial medical insurance is ¥9,032.353, which is higher than the average of households without commercial medical insurance, which is ¥8,750.217. The average value of risky financial assets to total financial assets of households with commercial medical insurance is 0.2625, which is much higher than the average value of households without commercial medical insurance, which is 0.0956. The mean value of total income of commercially insured households is ¥192,100.6, which is also much higher than that of non-commercially insured households at ¥91,126.71 (Table 7).

**Table 7:** Descriptive statistics by whether or not to purchase commercial medical insurance.

	Whether to participate in commercial medical insurance	
	No	Yes
Health risk	0.5053	0.3806
Household health risks	2.1459	1.5029
Medical Expenses	8,750.217	9,032.353
Risky assets ratio	0.0956	0.2625
Risky financial assets	0.4139	0.7056
Total income	91,126.71	192,100.6

In light of this, it is possible to assume that, in the first instance, commercial medical insurance may have the so-called “risk constraint effect,” which lowers home health risks and their consequences. Second, although the cost of premiums is higher for commercial medical insurance, households that acquire it often have higher income levels, thus premiums represent a smaller percentage of household income and have a smaller eroding effect. This, along with the higher level of coverage provided by commercial medical insurance, can effectively reduce the risk of significant medical expenses, which in turn can raise investors’ risk tolerance and encourage the allocation of hazardous financial assets.

## 4 Empirical analysis

### 4.1 Probit regression

This article uses the Probit model to verify the effect of health risk and social medical insurance on household financial asset allocation. From the regression results, the effect of health risk on household financial asset allocation is significantly negative at the 1% level, which shows that health risk reduces household participation in risky financial markets. Column (2) considers the effect of social medical insurance on household financial asset allocation with a coefficient of −0.0974, indicating that the effect of participation in social medical insurance on household holdings of risky financial assets is significantly negative. In other words, social medical insurance reduces the household’s willingness to hold risky financial assets. In order to test the hypothesis that social medical insurance reduces health risk and its marginal effects, column (3) introduces an interaction term between household health risk and participation in social medical insurance. The results show that the coefficient of the interaction term is significantly positive at the 1% level, 0.0614, which indicates that health-risk households with social medical insurance have a greater willingness to hold household risky financial assets than health-risk households without social medical insurance, and that medical insurance can significantly and positively increase the risk preferences of health-risk households. That is, medical insurance can weaken health risk and its marginal effect on the willingness to hold household risky financial assets. Hypothesis 1 is tested (Table 8).

**Table 8:** Baseline regression.

Risky financial assets	(1)	(2)	(3)
Household health risks	−0.114*** (−77.71)	−0.115*** (−78.11)	−0.169*** (−42.43)
social medical insurance		−0.0974*** (−11.85)	−0.238*** (−18.99)
Household health risks × social medical insurance			0.0614*** (14.78)
Control variables			
Numbers	−0.0196*** (−12.63)	−0.0197*** (−12.71)	−0.0193*** (−12.41)
Education	0.0160*** (37.31)	0.0161*** (37.55)	0.0165*** (38.23)
Age2	−0.00556*** (−45.83)	−0.00545*** (−44.76)	−0.00541*** (−44.44)
Ln_medical expenses	0.129*** (83.66)	0.129*** (83.65)	0.129*** (83.33)
Gender	0.0321*** (7.20)	0.0321*** (7.20)	0.0324*** (7.27)
Social pension insurance	0.0000831*** (23.51)	0.0000817*** (23.11)	0.0000813*** (22.99)
Ln_total income	0.0262*** (36.41)	0.0264*** (36.68)	0.0263*** (36.53)
_cons	−0.203*** (−15.90)	−0.118*** (−8.00)	0.00428 (0.25)
Size of sample	34,603	34,603	34,603
R^2^	0.0379	0.0382	0.0387

*t* statistics in parentheses; *p < 0.05, **p < 0.01, ***p < 0.001.

It makes intuitive sense that social medical insurance would lessen the financial strain that an insured person’s medical bills would place on their family, reducing the need for precautionary reserves and raising the preference for risk among financial assets. The Probit regression model’s findings, however, indicate that signing up for social medical insurance actually lowers household holdings of risky financial assets. This finding can be explained in the following ways:

First, China’s social medical insurance system is still in need of improvement. The majority of its insurance policies offer only modest coverage, particularly for major diseases, which has little impact on the cost of care for families and does not significantly improve large medical expenses. In other words, the substitution and risk-binding effects of social medical insurance are limited due to its insufficient coverage. Second, social medical insurance raises the insured expenditures of residents. As the cost of our health care system rises, social medical insurance premiums also rise annually, which further raises the general population’s health care costs. As a result, some households’ financial situations may worsen, decreasing their willingness to take on risk. In other words, social medical insurance has major consequences on risk compensation and erosion that will raise premium costs, lower disposable income, and consequently lower risk appetite among residents. Third, for some residents, enrolling in social medical insurance may make them lower their precautionary savings and increase their consumption, without improving their financial situation and shifting their capital to investment in risky financial assets. Therefore, due to the above factors, social medical insurance does not increase households’ risk appetite for financial assets, but rather decreases households’ willingness to hold risky financial assets.

### 4.2 Endogeneity analysis

The main explanatory variable of social medical insurance faces a reverse causality issue, hence the instrumental variables approach is used for testing in this research. There is a reverse causality issue with the selection of medical insurance. Families who invest more in hazardous financial assets are likely to be better off financially on their own, be more aware of the benefits of insurance, and spend less of their assets on insurance premiums. As a result, such households are more inclined to get insurance with more coverage at a higher price. Most researchers choose the indicator of participation rate when choosing the instrumental variable of medical insurance. The regional medical insurance participation rate is the instrumental variable in this article. The regional medical insurance participation rate is related to individual insurance participation behavior and might, to some extent, reflect the region’s citizens’ financial situation and insurance knowledge. This article calculated the regional social medical insurance participation rate as an instrumental variable.

According to the results, the Wald test rejected the original hypothesis of exogeneity of medical insurance at the 1% level of significance (Table 9). The *F*-value in the two-stage estimation method is also significant, corresponding to a value of *p* of 0.0000, so there is no weak instrumental variable. The findings demonstrate that household health risk decreases household hazardous financial asset allocation, with the effect of health risk being significantly negative at the 1% level. “Column (3) has been added to reflect the interaction between household health risk and social medical insurance in order to confirm the risk-contingent effects of social medical insurance”. The regression results in column (2) demonstrate that the effect of social medical insurance on household risky financial asset allocation is significantly negative. The findings indicate that social medical insurance has a risk-constrained effect that is significant at the 1% level. It agrees with the results of the initial regression analysis. However, the results of the instrumental variables approach differ from those of the benchmark regression in terms of regression coefficients, thus indicating that the endogeneity issue has an impact on the empirical results.

**Table 9:** Instrumental variable method: IV-probit regression.

	(1)	(2)	(3)
Household health risks	−0.109*** (−69.25)	−0.120*** (−78.91)	−0.408*** (−22.33)
Social medical insurance		−0.727*** (−17.64)	−1.514*** (−17.14)
Household health risks × social medical insurance			0.332*** (16.50)

Control variables	Control	Control	Control
Size of sample	34,603	34,603	34,603
One stage F test value of *p*	0.0000	0.0000	0.0000
Wald test Chi2	15,473.47	16,269.62	15,482.26
(*p*-value)	0.0000	0.0000	0.0000

*t* statistics in parentheses; *p < 0.05, **p < 0.01, ***p < 0.001.

### 4.3 Robustness test

#### 4.3.1 Logit model

The article is verified with a logit model to assess the outcomes of the baseline regression. According to the regression results, it can be seen that column (1) considers health risk with a coefficient of −0.187, and health risk still decreases household risky financial asset allocation at the 1% level of significance. Column (2) considers the effect of social medical insurance on whether residents purchase risky assets with a coefficient of −0.161, which is significant at the 1% level, indicating that social medical insurance discourages residents’ investment in risky assets. The interaction term between household health risk and social medical insurance is further introduced in (3), and the results suggest that medical insurance can reduce health risk and its marginal effects. Hypothesis 1 has been verified. Consistent with the findings of the benchmark regression, the model can therefore be considered robust (Table 10).

**Table 10:** Logit regression.

	(1)	(2)	(3)
Household health risks	−0.187*** (−77.27)	−0.188*** (−77.67)	−0.278*** (−41.02)
Social medical insurance		−0.161*** (−12.06)	−0.386*** (−18.78)
Household health risks × social medical insurance			0.101*** (14.32)

Control variables	Control	Control	Control
Size of sample	34,603	34,603	34,603
R2	0.0383	0.0387	0.0391

*t* statistics in parentheses; *p < 0.05, **p < 0.01, ***p < 0.001.

#### 4.3.2 Subjective assessment replaces objective indicators

In this article, a subjective indicator of whether people are willing to invest in hazardous financial assets replaces the objective indicator of whether the explanatory variable in the benchmark regression holds risky financial assets. After the variables are replaced, probit regressions are run. The results show that column (1) takes household health risk into account. The coefficient of the regression, which is close to −0.0836, is significant at the 1% level and shows how households’ willingness to hold risky financial assets is significantly reduced as a result. Column (2) considers the effect of social medical insurance on residents’ willingness to invest in risky financial assets with a positive coefficient, but later, after gradually adding the interaction term in column (3), the coefficient is significantly negative at the 1% level, so it can be assumed that social medical insurance reduces households’ willingness to invest in risky financial assets. Column (3) adds the interaction term between household health risk and social medical insurance, and the coefficient is positive (0.0228) and significant at the 1% level. Thus, it shows that social medical insurance significantly reduces the marginal effect of health risk on the willingness to invest in risky financial assets of the population. Hypothesis 1 is tested. It can be concluded from this that the benchmark regression results are robust (Table 11).

**Table 11:** Subjective assessment replaces objective indicators probit regression.

Willingness	(1)	(2)	(3)
Household health risks	−0.0836*** (−58.62)	−0.0834*** (−58.43)	−0.103*** (−30.46)
Social medical insurance		0.0217** (2.67)	−0.0357** (−2.96)
Household health risks × social medical insurance			0.0228*** (6.45)

Control variables	Control	Control	Control
Size of sample	34,603	34,603	34,603
R2	0.0192	0.0192	0.0193

*t* statistics in parentheses; *p < 0.05, **p < 0.01, ***p < 0.001.

#### 4.3.3 Ratio instead of value

In this article, the baseline regression’s explanatory variables for household health risk rate and household social medical insurance participation rate are substituted for the baseline regression’s explanatory variables for household health risk and household social medical insurance, respectively. A household’s level of health risk can be better estimated by looking at its household health risk rate. The impact of medical insurance on household risk financial market involvement is highlighted by the household participation rate, which more precisely reflects the household’s membership in social medical insurance. Household health risk considerably lowers household involvement rate, as shown by the Probit regression results. Column (2) considers the effect of household social medical insurance participation rate on whether to invest in risky financial assets with a coefficient of −0.426, which is significant at the 1% level, indicating that social medical insurance reduces household investment in risky financial assets. Column (3) adds the interaction term between health risk and social medical insurance, and the results indicate that social medical insurance increases the probability of investing in risky financial assets for healthy-risk households to some extent by acting on health risk, and reduces the marginal impact of health risk on households’ risky attitudes. The conclusions obtained are consistent with those of the benchmark regression and hypothesis 1 is tested, so the benchmark regression conclusions are robust (Table 12).

**Table 12:** Ratio instead of value probit regression.

	(1)	(2)	(3)
Household health risks	−0.427*** (−68.16)	−0.426*** (−67.83)	−0.602*** (−57.98)
Social medical insurance		−0.0270*** (−9.59)	−0.108*** (−22.87)
Household health risks × social medical insurance			0.154*** (21.34)

Control variables	Control	Control	Control
Size of sample	34,603	34,603	34,603
R2	0.0347	0.0349	0.0359

*t* statistics in parentheses; *p < 0.05, **p < 0.01, ***p < 0.001.

## 5 Heterogeneity analysis

### 5.1 Urban–rural heterogeneity

Given the stark disparities in household income, insurance philosophy, and quality of medical care between urban and rural locations, there may also be a disparity in how medical insurance affects the distribution of household financial assets. Therefore, this article is estimated separately for the rural and urban samples. According to the regression results, the effect of health risk on risky financial asset allocation of urban households is −0.132, which is much smaller than the inhibitory effect of −0.205 in rural areas. The effect of social medical insurance on urban residents −0.184 is also much smaller than the effect on rural residents −0.312. in addition, the effect of social medical insurance on reducing health risk and its marginal impact is better in rural areas (Table 13).

**Table 13:** Analysis of urban–rural heterogeneity.

	Urban	Rural
Household health risks	−0.132*** (−25.10)	−0.205*** (−31.50)
Social medical insurance	−0.184*** (−12.27)	−0.312*** (−13.35)
Household health risks × social medical insurance	0.0356*** (6.46)	0.0917*** (13.57)

Control variables	Control	Control
Size of sample	22,691	11,912
*R*^2^	0.0335	0.0328

*t* statistics in parentheses; *p < 0.05, **p < 0.01, ***p < 0.001.

The following factors may be the main causes: first, because income levels in rural areas are uncertain and residents have less sophisticated investment and financial concepts, they are more risk-averse and save more money. Additionally, the eroding effect of social medical insurance is significant in rural areas where medical insurance payments represent a bigger percentage of inhabitants’ income. These traits cause rural populations to adopt more cautious investment strategies and to be more sensitive to risky financial assets. Thus, it can be concluded that in rural areas, residents are more responsive to household health risks. Social medical insurance has a stronger disincentive effect on holding risky financial assets in households. Second, because people in rural areas tend to be less health-conscious, social medical insurance can better emphasize the value of health to locals. Additionally, medical insurance can lower the cost of medical care, promoting rural inhabitants’ access to more medical services, enhancing their health, and increasing their likelihood of curing themselves. In contrast, social medical insurance has less of an impact on raising health risks in urban areas with more robust medical infrastructure and public health awareness. Social medical insurance, therefore has a larger risk-binding effect in rural areas, which can effectively minimize health risk and its marginal impact as well as encourage rural residents’ ownership of risky financial assets.

### 5.2 Regional heterogeneity

Since the reform and opening up, the gap in economic development between the less developed and developed^1^ regions of China has tended to increase. This article considers the differences between developed and less developed regions and regresses them into groups. According to the regression results, it can be obtained that, overall, the inhibitory effect of health risk on household risk preferences is more pronounced in less developed regions, with a coefficient of −0.217, which is larger than that of −0.116 in developed regions. In the less developed regions, social medical insurance also has a more pronounced suppressive effect on household risk preferences, with a coefficient of −0.282, which is larger than that of −0.216 in the developed regions, mainly because: in the less developed regions, the level of economic development is more backward, most residents have limited income levels, and premiums account for a high proportion of household income. Therefore, the erosive effect of social medical insurance is more substantial, and residents prefer less risky financial assets such as cash and deposits. Additionally, social medical insurance has a more significant role in preventing the allocation of financial assets in less developed countries where individuals tend to have conservative investment views and are fundamentally less tolerant of risky assets. The marginal effect of medical insurance is more potent in less developed regions, hence the interaction term’s coefficient in less developed regions is 0.0802 rather than 0.0451 in developed countries. Medical insurance can significantly boost access to healthcare, thereby enhancing the health of people living in less developed areas, which in turn increases their willingness to take risks. Hypothesis 2 is tested (Table 14).

**Table 14:** Analysis of regional heterogeneity.

	Developed area	Underdeveloped area
Household health risks	−0.116*** (−20.48)	−0.217*** (−38.33)
Social medical insurance	−0.216*** (−12.60)	−0.282*** (−15.33)
Household health risks × social medical insurance	0.0451*** (7.60)	0.0802*** (13.65)

Control variables	Control	Control
Size of sample	16,768	17,835
R^2^	0.0359	0.0436

*t* statistics in parentheses; *p < 0.05, **p < 0.01, ***p < 0.001.

### 5.3 Household head gender heterogeneity

The household’s involvement in insurance might be influenced by the head of the household because they have a significant role in the home. Additionally, the head of the home typically has a higher economic status within the family, and the head of the household’s attitude toward risk influences the family’s ownership of risky financial assets. As a result, the article is separated into sections based on the gender of the household head and estimates for male and female household heads are done separately. The empirical results indicate that the negative impact of health risk on female heads of household(−0.166) is smaller than that on male heads of household(−0.172). The impact of social medical insurance on the suppression of risk preference of female heads of household is smaller −0.212 than that of male heads of household −0.262, both significant at the 1% level. The coefficient of 0.0572 for female heads of households is smaller than that of 0.0653 for male heads of households, probably because women are more risk-averse and prefer to choose low-risk products in the asset selection process. Therefore, for female heads of households, they tend to choose low-risk products regardless of whether they purchase social medical insurance or not, and the substitution effect and risk constraint effect of social medical insurance is weaker (Table 15).

**Table 15:** Analysis of gender heterogeneity of household heads.

	Male	Female
Household health risks	−0.172*** (−30.61)	−0.166*** (−29.34)
Social medical insurance	−0.262*** (−14.93)	−0.212*** (−11.88)
Household health risks × social medical insurance	0.0653*** (11.15)	0.0572*** (9.72)

Control variables	Control	Control
Size of sample	28,450	7,364
R^2^	0.0385	0.0389

*t* statistics in parentheses; *p < 0.05, **p < 0.01, ***p < 0.001.

### 5.4 Household head age heterogeneity

The middle-aged and older adult samples are estimated separately based on the age of the household head, which is divided accordingly. According to Chinese tradition, those between the ages of 16 and 60 are considered middle-aged and young, while those over 60 are considered older adult. The empirical results show that the effect of health risk is much larger for young and middle-aged households −0.180 than for older households −0.0951. The coefficient of the effect of participation in social medical insurance on whether young and middle-aged households hold risky assets is −0.228, which is larger than that of older households −0.145. The risk constraint effect of medical insurance is also stronger for middle-aged and young-headed households than for older-headed households, and the coefficient of the interaction term for older-headed households is not significant. It can be seen that the effect of medical insurance on the willingness of the older adult to hold risky financial assets is small (Tables 16).

**Table 16:** Analysis of age heterogeneity of household heads.

	Young and middle-aged	Aged
Household health risks	−0.180*** (−34.01)	−0.0951*** (−9.06)
Social medical insurance	−0.228*** (−13.80)	−0.145*** (−4.61)
Household health risks × social medical insurance	0.0863*** (15.66)	0.0159 (1.48)

Control variables	Control	Control
Size of sample	20,232	14,363
R^2^	0.0515	0.0242

*t* statistics in parentheses; *p < 0.05, **p < 0.01, ***p < 0.001.

The descriptive statistics show that while the participation rate of social medical insurance is higher for the older adult than for the young and middle-aged, it is much lower for commercial medical insurance, which offers a higher level of coverage. It could be that the older adult were unaware of insurance in the early stages and lost the best opportunity to insure because China’s commercial medical insurance did not launch until much later and did not have a robust protection mechanism at the outset. In addition, the investment intention and holding ratio of risky financial assets of the older adult are much different from those of the middle-aged and young people; due to their age, the physical condition of the older adult is weaker than that of the middle-aged and young people, which corresponds to higher medical expenses. Combined with the analysis, it can be concluded that the reasons for the above regression results may be: first, the older adult themselves have limited investment concepts and are less receptive to risky financial assets, so their willingness to invest in risky investments is lower whether they hold medical insurance or not; Second, older adults are less physically fit and therefore need more precautionary savings to prevent unanticipated medical expenditures, and will adopt more risk-averse behavior regardless of whether they are enrolled in Medicare or not. Therefore, medical insurance has less impact on the allocation of risky financial assets among elderly-headed households.

## 6 The role of insurance coverage level: the difference between social medical insurance and commercial medical insurance

This article will further examine the influence of two significant insurance systems with various levels of coverage, social medical insurance and commercial medical insurance, in order to investigate the varying effects of the level of medical insurance coverage on family financial asset allocation. Social and private medical insurance make up the majority of China’s present medical insurance system. Commercial medical insurance refers to an insurance policy in which the policyholder pays a specified premium according to the contract. When the insured person incurs medical expenses due to illness or accidents, the insurer provides compensation or pays out insurance benefits in accordance with a predetermined percentage. Commercial medical insurance premiums are typically higher, and households that enroll in commercial medical insurance tend to have better financial conditions. The impact of premiums on their income is usually minimal. Commercial insurance offers a wider range of coverage, including general medical insurance, accidental injury medical insurance, hospitalization medical insurance, surgical medical insurance, and specialized disease insurance. These insurance types provide compensation for various risks from different perspectives and at different proportions. Typically, families that enroll in commercial medical insurance also tend to have social medical insurance simultaneously, thereby attaining a more comprehensive level of healthcare security. In contrast, social medical insurance plans are relatively limited in terms of coverage options compared to commercial medical insurance. Therefore, households still need to bear some medical expenses themselves. Moreover, the majority of participants in social medical insurance are enrolled in basic medical insurance schemes, such as resident medical insurance, which offers lower coverage levels. These plans primarily target urban and rural families with unstable income sources, and the premiums are significantly lower, reducing disposable income. Different medical insurance plans offer varying degrees of coverage for unforeseen medical costs (53), and as a result, they have varying effects on a household’s risk tolerance. In light of this, an empirical study is conducted in this work, and the outcomes are as follows (Table 17).

**Table 17:** Descriptive statistics by age.

Mean	Young and middle-aged	Aged
Social medical insurance	0.9182	0.9475
Commercial medical insurance	0.0328	0.0084
Risky financial assets	0.4286	0.3737
Risky assets ratio	0.1023	0.07990
Health risk	0.4390	0.6836
Medical expenses	7,782.536	11,139.99

Medical insurance	Yes	No	Yes	No
Risky financial assets	0.4300	0.4134	0.3729	0.3871
Health risk	0.4266	0.5814	0.6790	0.7655

*t* statistics in parentheses; *p < 0.05, **p < 0.01, ***p < 0.001.

This article uses a Probit model to test the effect of different types of medical insurance on household financial asset allocation (Table 18). Column (1) considers the effect of social medical insurance on household financial asset allocation with a coefficient of −0.0974, indicating that the effect of participation in social medical insurance on household holdings of risky financial assets is significantly negative, and social medical insurance reduces households’ willingness to hold risky financial assets. Column (2) considers the effect of commercial medical insurance with a coefficient of 0.617, which shows that the effect of participation in commercial medical insurance on households’ risky financial assets is significantly positive and the coefficient remains above 0.6, implying that participation in commercial medical insurance can significantly increase the risk appetite of residents. Column (3) considers the effects of both social and commercial medical insurance and obtains the same conclusion, and both are significant at the 1% level. In columns (4–6), the interaction term between household health risk and participation in medical insurance is added in order to test the hypothesis that medical insurance can reduce health risk and its marginal impact. The findings demonstrate that the interaction terms’ coefficients are significantly positive, indicating that households with health risks who have medical insurance are more likely than households without medical insurance to hold household risky financial assets. These findings also demonstrate that the interaction terms’ coefficients are significantly positive in raising household risk preferences. That is, medical insurance can weaken health risk and its marginal impact on the willingness to hold risky financial assets of households. The interaction term coefficient of commercial medical insurance is larger than that of social medical insurance, which means that the marginal effect of health risk is weakened more by commercial medical insurance than by social medical insurance, which can also indicate that medical insurance with higher coverage can better increase the allocation of household risky financial assets. Hypothesis 3 is tested.

**Table 18:** The probit regression of commercial medical insurance and social medical insurance.

	(1)	(2)	(3)	(4)	(5)	(6)
Household health risks	−0.115*** (−78.11)	−0.113*** (−76.69)	−0.113*** (−77.10)	−0.169*** (−42.43)	−0.171*** (−42.90)	−0.167*** (−41.89)
Social medical insurance	−0.0974*** (−11.85)		−0.100*** (−12.17)	−0.238*** (−18.99)	−0.113*** (−75.94)	−0.239*** (−19.08)
Commercial medical insurance		0.617*** (43.93)	0.618*** (44.01)		0.625*** (30.75)	0.627*** (30.83)
Household health risks × Social medical insurance				0.0614*** (14.78)		0.0608*** (14.63)
Household health risks × Commercial medical insurance					0.199*** (31.53)	0.596* (0.65)

Control variables	Control	Control	Control	Control	Control	Control
Size of sample	34,603	34,603	34,603	34,603	34,603	34,603
R^2^	0.0382	0.0423	0.0426	0.0387	0.0409	0.0431

*t* statistics in parentheses; *p < 0.05, **p < 0.01, ***p < 0.001.

## 7 Conclusions and implications

### 7.1 Conclusions

In the financial work, improving the structure of household financial asset allocation, nearby residents’ consumption and promoting the development of investment and financing markets are important work tasks. From the available literature, most of them are developed from life cycle, demographic characteristics, etc. This article focuses on the effects of medical insurance and health risks on household financial asset allocation. The impact of medical insurance is analyzed in four aspects: substitution effect, erosion effect, risk compensation effect, and risk constraint effect. The impact of health risk is analyzed in terms of its effect on expenditure, income and savings. This article analyzes data based on the China Household Financial Survey (CHFS) 2015–2019, and draws the following conclusions: 1. Health risk, as an important background risk for households, may increase households’ unanticipated medical expenditures, reduce household income sources, and households will therefore increase precautionary savings, thus further reducing their investments in risky financial assets; 2. As a medical insurance to diversify the risk of household unanticipated medical expenditures, social medical insurance with a lower degree of coverage produces a weaker substitution effect, which is affected by the erosion effect, etc. It will instead reduce household risk appetite and reduce the allocation of household risky financial assets. However, the prevalence of social medical insurance draws attention to health. Households covered by medical insurance have a higher probability of focusing on health maintenance, seeking timely medical treatment, and improving their health. Thus medical insurance produces a risk suppression effect that can increase risky financial asset allocation by reducing health risk and its marginal impact, thus improving household risk-taking capacity. 3. Urban households, households in developed areas, male-headed households, and middle-aged and young-headed households have higher risk appetite and are more likely to make risky investments, and such households are more risk-sensitive and therefore more affected by health risks and social medical insurance. 4. Medical insurance can have a heterogeneous impact on household financial asset allocation due to different levels of coverage. Commercial medical insurance can significantly increase household risk financial market participation because of its higher level of coverage, which can better reduce the risk of unexpected household medical expenses. It can also better reduce the marginal impact of health risks on household financial asset allocation.

### 7.2 Policy implications

The conclusion of this article shows that health risk can inhibit participation of household risky financial market, while medical insurance can reduce the marginal impact of health risk, and the higher coverage of medical insurance can promote the allocation of household risky financial assets. Therefore, in order to improve the current situation of high saving rate of residents in developing countries such as China, and promote residents’ investment and consumption, this article proposes the following recommendations:

First, the government can implement policies to incentivize residents in rural and underdeveloped areas to purchase health insurance. These policies may encompass providing fiscal subsidies, appropriately reducing insurance costs, and expanding insurance coverage. Such measures are expected to contribute to an increase in the adoption rate of health insurance, particularly in regions with sparse populations or limited economic resources. This, in turn, would better shield households from the financial burdens associated with health risks and enhance their participation in financial markets.

Secondly, use commercial medical insurance, improve industry regulation, and raise awareness of commercial medical insurance. Due to a lack of knowledge about commercial medical insurance, the fragmented character of the commercial medical insurance market, and residents’ concern over the difficulties of making a compensation claim, there is currently a low participation rate of Chinese residents in commercial medical insurance. The appropriate authorities should promote commercial medical insurance more widely, speed up payouts more quickly, and strengthen oversight of the disarray in the commercial medical insurance market. The government and businesses must collaborate to further popularize pertinent insurance knowledge and educate families about medical insurance.

Create a top-notch, multi-tiered medical insurance scheme. The system has three levels. The first level is to create a high-quality, universal basic medical insurance program and address the issue of provincial cooperation. It emphasizes fairness, ensuring that residents and employees receive the same care, and covers the fundamental treatments for serious disorders. In order to address the issue of equality brought on by the significant economic disparities between urban and rural areas as well as across different regions, local governments, units, or businesses must provide standard supplementary medical insurance at the second level. In order to address residents’ needs, the third level is commercial medical insurance offered by commercial insurance companies, which is entirely purchased by individuals’ own choice.

## Data availability statement

Publicly available datasets were analyzed in this article. This data can be found here: https://chfs.swufe.edu.cn/.

## Author contributions

CL: Conceptualization, Formal analysis, Supervision, Writing – original draft, Writing – review & editing. JL: Data curation, Investigation, Methodology, Visualization, Writing – original draft, Writing – review & editing. CZ: Conceptualization, Methodology, Supervision, Validation, Visualization, Writing – review & editing. XD: Investigation, Software, Validation, Writing – review & editing. ZJ: Data curation, Methodology, Visualization, Writing – review & editing. SJ: Funding acquisition, Project administration, Resources, Supervision, Writing – review & editing.

## Footnotes

^1^Less developed regions are those regions that have certain economic strength and potential but still have certain gaps with developed regions, unbalanced productivity development, and underdeveloped science and technology levels, such as the central and western regions of China. Developed regions are those with more advanced economic and scientific development, such as the eastern region of China. According to China’s health statistics yearbook used to divide the east, middle and west: the eastern region including Beijing, Tianjin, Hebei, Liaoning, Shanghai, Jiangsu, Zhejiang, Fujian, Shandong, Guangdong, Hainan 11 provinces (municipalities directly under the Central Government), the central region including Heilongjiang, Jilin, Shanxi, Anhui, Jiangxi, Henan, Hubei, Hunan 8 provinces, the western region including Inner Mongolia, Guangxi, Chongqing, Sichuan, Guizhou, Yunnan, Tibet Shaanxi, Gansu, Qinghai, Ningxia, Xinjiang 12 provinces (autonomous regions and municipalities directly under the Central Government).
